# Oral Hygiene Interventions and Pneumonia Prevention in Critical Care: An Integrative Review of Evidence and Practice

**DOI:** 10.1155/ijod/4436498

**Published:** 2026-06-24

**Authors:** Chris Sara Mathew, Manjush Karthika, Khalid Ansari

**Affiliations:** ^1^ College of Medical and Health Sciences, Liwa University, Abu Dhabi, UAE

**Keywords:** critical care, non-ventilator hospital-acquired pneumonia, oral hygiene, patient safety, ventilator-associated pneumonia

## Abstract

**Background:**

Ventilator‐associated pneumonia (VAP) and non‐ventilator hospital‐acquired pneumonia (NVHAP) remain among the most common and serious infections in adult critical care settings. Despite strong biological plausibility, oral care is inconsistently specified and rarely measured as a preventive strategy. This review synthesizes evidence on oral hygiene practices in critically ill adults, examines their relationship to VAP and NVHAP, and proposes a pragmatic and dentistry‐informed oral‐care bundle for clinical use.

**Methods:**

An integrative review was conducted using a structured literature search in PubMed, Scopus, and Web of Science from 2005 to 2025. Search terms combined pneumonia‐related concepts, critical care setting and oral health. Eligible sources included studies addressing oral health and the prevention of VAP or NVHAP in adult critical care settings. Only full‐text publications in English or with an available English translation were included.

**Results:**

The database search identified 935 records. After removing 224 duplicates, 711 studies were selected for title and abstract screening. Furthermore, studies focused exclusively on non‐oral pneumonia prevention strategies, those without an oral health‐related component, or those not conducted in an acute hospital or intensive care unit (ICU) setting were excluded during the synthesis phase, resulting in 20 studies included in the final synthesis. The evidence synthesis was conducted narratively, organized around domains corresponding to the review objectives such as pathophysiologic mechanisms along the oral–lung axis; current oral care practices; modality‐specific interventions; assessment and documentation; implementation and workforce factors; safety, equity, and feasibility.

**Conclusion:**

Oral hygiene occupies a modifiable point along the oral–lung axis, where simple bedside interventions can reduce pneumonia risk; however, clinical practice remains heterogeneous and under‐specified. Structured oral care practices reduce VAP incidence and represent a reliable and scalable contribution to safer critical and acute care.

## 1. Introduction

Ventilator‐associated pneumonia (VAP) and non‐ventilator hospital‐acquired pneumonia (NVHAP) remain among the most common serious infections in adult acute care and critical care. VAP is reported to affect 5%–40% of patients receiving invasive mechanical ventilation for more than 48 h, and overall mortality rates of around 30% have been reported in patients with NVHAP [1, 2]. NVHAP affects approximately 1 in every 100 hospitalized patients, has a crude mortality rate of 15%–30%, extends hospital length of stay (LOS) by up to 15 days, requires intensive care unit (ICU) admission in up to 46% of non‐ICU cases, increases antibiotic utilization, and is associated with readmission within 30 days in up to 20% of survivors [3]. VAP carries a measurable attributable ICU mortality, largely mediated through prolonged mechanical ventilation and LOS [4]. NVHAP is now recognized as at least as frequent as VAP, contributing to sepsis, ICU transfer, and discharge to institutional care, yet it is often unmeasured and unreported in routine surveillance [5, 6].

Across both VAP and NVHAP, there is a consistent biological link between the oral cavity and the respiratory system. Under conditions of critical illness, sedation, and mechanical ventilation, dental plaque and oropharyngeal biofilm shift from largely commensal flora toward gram‐negative bacilli and other respiratory pathogens within 48 h of ICU admission. These communities colonize teeth, gingiva, tongue, dentures, endotracheal tubes, and oropharyngeal devices and can be transported into the lower respiratory tract through micro aspiration or macro aspiration events [7–9]. Impaired cough reflex, reduced salivary flow, prolonged supine positioning, and endotracheal tube cuff leak collectively compromise airway protective mechanisms, thereby facilitating aspiration of the pathogenic biofilm into the lower respiratory tract and increasing the risk of pneumonia development.

Oral hygiene is, therefore, more than a comfort measure. Randomized controlled trials, systematic reviews, and network meta‐analyses indicate that structured oral care regimens are associated with a reduction in VAP incidence, while effects on mortality, overall LOS, and duration of mechanical ventilation are less consistent and remain uncertain [9–11]. NVHAP initiatives from the Agency for Healthcare Research and Quality (AHRQ) have emphasized oral care as a core component of successful VAP prevention bundles while also highlighting substantial gaps in implementation and documentation [12]. Despite a growing body of evidence, oral care practices in real‐world ICUs remain inconsistent. Surveys consistently report inconsistent toothbrushing, frequent reliance on foam swabs, partial protocol adherence, and limited use of structured oral assessment tools [13–15].

This integrative review focused on oral care and the prevention of VAP and NVHAP in adult critical care settings and was guided by the following specific objectives: (1) to examine pathophysiologic mechanisms and modifiable targets along the oral–lung axis; (2) to describe current oral care practices in ICUs; (3) to synthesize comparative evidence on key oral care methods, including toothbrushing, antiseptics, suctioning, moisturization, and device care and propose a pragmatic, dentistry‐informed oral‐care bundle for clinical use; (4) to identify challenges related to assessment, implementation, documentation, and staffing; to highlight persistent knowledge gaps and priority areas for future research.

## 2. Methods

This review was conducted using an integrative review methodology as it allows the inclusion and synthesis of diverse types of evidence, including experimental studies, observational studies, and clinical practice literature. This approach was considered appropriate for comprehensively examining the range of oral care interventions, implementation practices, and evidence gaps related to pneumonia prevention in hospitalized patients.

A structured literature search was conducted in PubMed, Scopus, and Web of Science from 2005 to 2025, using a combination of keywords, Medical Subject Headings (MeSHs) terms, and Boolean operators (AND/OR). This date range was chosen to focus on the growing number of publications on the subject over the last 20 years. Search terms combined pneumonia‐related concepts, critical care setting, and oral health.

Search terms “ventilator associated pneumonia,” “non‐ventilator hospital acquired pneumonia,” “hospital acquired pneumonia,” “aspiration pneumonia,” “critical care,” “intensive care units,” “mechanical ventilation,” “subglottic suction,” “oral health,” “oral hygiene,” “dental health,” “oral care practices,” and “oral hygiene practices” were used. The search was supplemented by manual screening of reference lists from the included studies. The search strategy was developed collaboratively by all three authors to ensure the comprehensive identification of all relevant studies. In addition, seminal and foundational studies published outside the predefined database year limits were retained for background context in the Sections 1 and 4.

A standardized data‐extraction form captured study design, setting, population, oral‐care components (mechanical cleaning, antiseptic, suction, moisturization, and device/denture care), outcomes (VAP/NVHAP incidence, mortality, LOS, and duration of mechanical ventilation), implementation factors, and adverse events. Eligible sources included randomized and quasi‐experimental studies, observational research, systematic and network meta‐analyses, implementation studies, and clinical or organizational guidance addressing oral health and the prevention of VAP or NVHAP in adult critical care settings. Only full‐text publications in English or with an available English translation were included. Pediatric studies, case reports, and papers focused exclusively on non‐oral pneumonia prevention strategies were excluded.

Screening of the studies was done independently by authors (Chris Sara Mathew and Manjush Karthika), who reviewed the titles, abstracts, and full‐text articles. Any disagreements during the screening process were resolved through discussion between the reviewers (Chris Sara Mathew and Manjush Karthika), and where necessary, consultation with a third reviewer was undertaken to reach a final decision (Khalid Ansari). To ensure a structured and transparent appraisal of heterogeneous evidence, we applied the American Association of Critical‐Care Nurses (AACN) Evidence‐Level Hierarchy, a framework that categorizes evidence from Level A (meta‐analyses and systematic reviews of randomized controlled trials) to Level E (expert opinion) [16, 17]. AACN evidence levels were used to classify the strength of evidence rather than methodological risk of bias, and disagreements in classification were resolved by consensus between reviewers. In the present review, this framework supported a rigorous and consistent evaluation of the included studies.

## 3. Results

**Figure 1:**
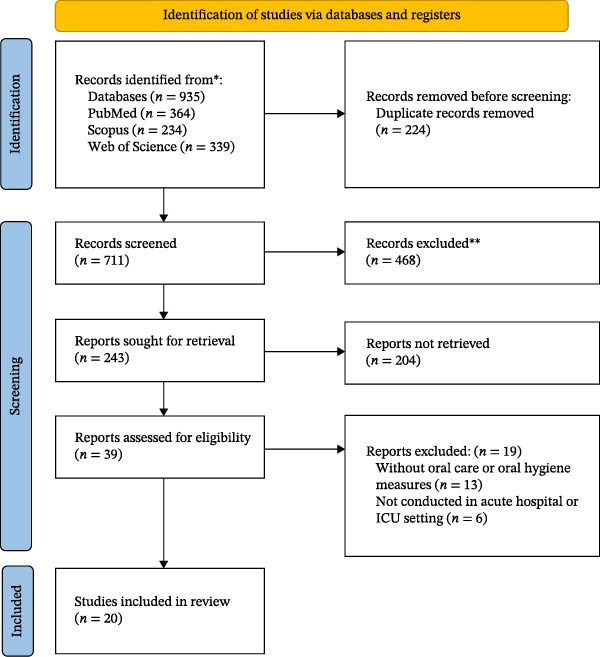
The PRISMA 2020 flowchart for the literature search process.

The search yielded 935 studies. After removing 224 duplicates, 711 studies underwent title and abstract screening. Of these, 468 studies were excluded, leaving 243 studies for full‐text review. Subsequently, 204 articles that did not meet the eligibility criteria were excluded, resulting in 39 studies. Furthermore, studies focused exclusively on non‐oral pneumonia prevention strategies without an oral health‐related component or those not conducted in acute hospital or ICU settings were excluded during the data synthesis phase. Consequently, 20 studies were included in the final data synthesis (Figure 1).

The studies included were categorized by strength of evidence using the AACN evidence‐level hierarchy [16]. The new AACN framework has six rating levels. Meta‐analyses and meta‐syntheses are considered the highest level of evidence (Level A). Level B encompasses both randomized and non‐randomized well‐designed controlled experiments. Qualitative studies, descriptive or correlational studies, integrative reviews, systematic reviews, and randomized controlled trials with conflicting outcomes all fall under Level C. Level D refers to the peer‐reviewed professional organizational standards. Level E represents theory‐based evidence from multiple case reports and expert opinion, whereas Level M identifies manufacturer recommendations.

In the current review, nine of the 20 studies are at level C (45%), whereas seven are at level A (35%). The level of evidence for each included study is presented in Table 1.

**Table 1:** Summary of evidence informing oral‐care interventions for prevention of VAP and NVHAP.

Author, year	Study design	Population/setting	Oral care intervention	Comparator	Pneumonia‐related outcome	Key findings	Level of evidence
Berry et al., 2011 [10]	Randomized controlled trial	Adult ICU, mechanically ventilated	Standardized oral hygiene including toothbrushing plus different mouth‐rinse regimens	Alternative standardized mouth‐rinse regimens	Dental plaque colonization; VAP (secondary)	No significant differences in plaque colonization or VAP between groups; supports biological plausibility of mechanical oral care but not powered for VAP outcomes	Level B
Lorente et al., 2012 [18]	Randomized controlled trial	Adult ICU, ventilated	Toothbrushing + routine care	Routine care without brushing	VAP incidence	No significant difference in VAP incidence between groups (9.7% vs. 11.0%; OR 0.87, 95% CI 0.47–1.62; *p* = 0.75). Adding manual toothbrushing to chlorhexidine did not reduce VAP	Level B
de Lacerda Vidal et al., 2017 [19]	Randomized controlled trial	Adult ICU, mechanically ventilated	Toothbrushing plus 0.12% chlorhexidine gel	0.12% chlorhexidine without toothbrushing	VAP incidence; ventilation duration	Toothbrushing showed a nonsignificant trend toward lower VAP incidence (*p* = 0.084) and a significant reduction in duration of mechanical ventilation; no significant effect on ICU stay or mortality	Level B
Zhao et al., 2020 [9]	Systematic review and meta‐analysis	Critically ill adults on mechanical ventilation	Oral hygiene care including chlorhexidine and/or toothbrushing (±suction)	Placebo or usual care	VAP incidence; mortality; ICU outcomes	Moderate‐certainty evidence shows chlorhexidine as part of oral care reduces VAP incidence; toothbrushing (±antiseptics) may reduce VAP and ICU stay (low certainty). No consistent effects on mortality, ventilation duration, or ICU length of stay	Level A
Klompas et al., 2014 [20]	Systematic review and meta‐analysis	Adult patients on mechanical ventilation (cardiac and noncardiac surgery)	Chlorhexidine oral care	Placebo or usual care	VAP; mortality; ICU outcomes	Chlorhexidine reduced respiratory infections in cardiac surgery patients but did not reduce VAP in noncardiac surgery patients. No benefit on mortality, ventilation duration, or ICU stay; possible signal toward increased mortality in noncardiac surgery cohorts	Level A
Yamakita et al., 2024 [11]	Network meta‐analysis	Adult ICU patients on invasive mechanical ventilation	Brushing ± chlorhexidine (0.12%); antiseptics alone	Other oral hygiene methods	VAP incidence	Brushing combined with chlorhexidine ranked highest for VAP prevention, followed by chlorhexidine alone and brushing alone; however, confidence in all comparisons was low, and further research is required	Level A
Kelly et al., 2023 [21]	National point‐prevalence study	Adult ICUs (U.K.)	Observed routine oral care practices	N/A	Oral care delivery (context for VAP/NVHAP prevention)	Oral care practices were highly variable; only 41% received toothbrushing and 44% had documented oral assessment. Limited protocol use and inconsistent assessment highlight major implementation gaps	Level C
Baker and Quinn, 2018 [22]	Multicenter retrospective chart review	Adult acute care hospitals (U.S.)	Documented pneumonia‐prevention practices (including oral care)	Absence of documented preventive care	NVHAP incidence and burden	NVHAP occurred across hospital units, predominantly outside ICUs, and was frequently preceded by absence of basic preventive care, highlighting major gaps in routine pneumonia‐prevention practices	Level C
Lacerna et al., 2020 [23]	Prospective observational study	All hospitalized patients across 21 hospitals (U.S.)	Multicomponent HAP prevention bundle including oral care	Preintervention baseline	HAP incidence, mortality, antibiotic use	Implementation of a hospital‐wide prevention program was associated with significant reductions in HAP rates, HAP‐related mortality, and broad‐spectrum antibiotic use; oral care was a core component alongside other basic nursing interventions	Level C
Livesey et al., 2024 [24]	Narrative review	Acute and nonventilated adult inpatients	Oral hygiene interventions discussed (among other NVHAP prevention strategies)	N/A	NVHAP incidence and prevention context	Oral hygiene is identified as a key NVHAP preventive strategy within broader non‐ventilator pneumonia prevention practices, but the evidence base is weak and heterogeneous	Level E
Causey et al., 2023 [25]	Scoping review	Adults mechanically ventilated at initiation (<48 h)	Quantitative oral health assessment tools	N/A	Oral health status (context for VAP risk)	Identified wide variation and limited standardization in oral health assessment methods at intubation, limiting comparability across studies; highlights need for validated, standardized oral assessment tools to inform targeted oral care and VAP prevention	Level C
Han et al., 2025 [12]	Rapid review	Adult inpatients (nonventilated)	Oral care protocols within PSPs and multicomponent bundles	Usual care or preintervention baseline	NVHAP incidence	Evidence for NVHAP prevention is limited and inconclusive. Prevention bundles (all including oral care) showed possible benefit (low strength of evidence), while evidence for oral care alone and other PSPs was insufficient due to study limitations and imprecision	Level C
Gu et al., 2012 [26]	Systematic review and meta‐analysis	Adult ICU patients on mechanical ventilation	Oral care including toothbrushing	Oral care without toothbrushing	VAP incidence; ICU mortality; ventilation duration; ICU length of stay	Toothbrushing did not significantly reduce VAP incidence (RR 0.77, 95% CI 0.50–1.21), ICU mortality, duration of mechanical ventilation, or ICU length of stay. Evidence was limited to four small trials, and authors emphasized the need for larger, well‐designed RCTs	Level A
Chan et al., 2007 [27]	Systematic review and meta‐analysis	Mechanically ventilated adult ICU patients	Daily oral decontamination with antiseptics or topical antibiotics	No oral decontamination	VAP incidence; mortality; ventilation duration; ICU length of stay	Oral decontamination with antiseptics significantly reduced VAP incidence (RR 0.56, 95% CI 0.39–0.81), whereas topical antibiotics did not. Neither strategy reduced mortality, duration of mechanical ventilation, or ICU length of stay. Findings support reduction in VAP incidence without evidence of benefit on mortality, duration of mechanical ventilation, or ICU length of stay	Level A
Deschepper et al., 2018 [28]	Hospital‐wide retrospective observational cohort study	Adult hospitalized patients (ICU and non‐ICU)	Chlorhexidine oral care (≤300 mg vs. >300 mg cumulative exposure)	No chlorhexidine exposure	Hospital mortality (safety outcome)	Chlorhexidine oral care was associated with increased hospital mortality in non‐ICU, nonventilated patients after adjustment (OR 2.61, 95% CI 2.32–2.92), with the strongest association in patients at lower baseline mortality risk. No harmful association was observed in ICU or mechanically ventilated patients. Findings argue against indiscriminate routine use of chlorhexidine oral care in hospitalized populations without proven benefit	Level C
De Cassai et al., 2024 [29]	Systematic review and network meta‐analysis	Adult ICU patients undergoing invasive mechanical ventilation	Oral chlorhexidine at any concentration	Placebo, sham intervention, usual care, or no intervention	VAP incidence; mortality; ventilation duration; ICU and hospital length of stay	Across all concentrations, chlorhexidine did not significantly reduce VAP incidence, mortality, duration of mechanical ventilation, or ICU or hospital length of stay. The authors conclude that routine use of chlorhexidine for VAP prevention in critically ill adults cannot be recommended	Level A
He et al., 2025 [30]	Systematic review and network meta‐analysis	Adult ICU patients receiving invasive mechanical ventilation	Mouthwash solutions including saline, chlorhexidine, povidone–iodine, oxidizing agents, sodium bicarbonate, and herbal extracts	No mouthwash or alternative solutions	VAP incidence; ICU mortality; duration of mechanical ventilation	Across 14 RCTs (*n* = 1644), no mouthwash consistently reduced VAP incidence or duration of mechanical ventilation. Oxidizing agents showed a nonsignificant trend toward lower VAP incidence, while saline solution was associated with reduced ICU mortality. Chlorhexidine did not demonstrate benefit on major outcomes and was associated with potential safety concerns. Evidence certainty was limited, and larger high‐quality trials were recommended	Level A
Tesoro et al., 2018 [31]	Retrospective observational chart review	Adult acute care inpatients (single tertiary hospital)	Routine nursing preventive care, including oral care (documentation review)	Missed or undocumented preventive care	NVHAP incidence; ICU transfer; discharge disposition; cost	NVHAP incidence was 0.47 per 1000 patient‐days, with substantial excess costs. Oral care was undocumented in 60.5% of cases, and NVHAP was associated with ICU transfer, increased post‐discharge care needs, and higher costs, highlighting missed basic nursing care as a major preventable factor	Level C
Stevenson et al., 2023 [32]	Concurrent mixed‐methods implementation study	Adult medical‐surgical and extended care units in six high‐complexity VA hospitals	Nurse‐led NVHAP prevention initiative (HAPPEN) including oral care education, supplies, and implementation support	Preimplementation practice (implicit)	NVHAP prevention context; implementation processes	Implementation barriers included sustaining oral care supplies and completing documentation, while facilitators included engaged nurse leadership, staff teamwork, and shared beliefs about infection prevention. Oral care was reframed as an essential infection‐control practice rather than a task. Lessons supported successful replication of the program nationwide and informed policy and practice	Level C
Ory et al., 2018 [33]	Concurrent mixed‐methods implementation study	Adult ICU patients undergoing mechanical ventilation across five ICUs	Oral care program incorporating toothbrushing with aspiration	Foam stick‐based oral care	VAP incidence; cost of care	Introduction of a toothbrushing‐with‐aspiration oral care program was associated with a significant reduction in VAP incidence (12.8% to 8.5%, *p* = 0.002) and substantial net cost savings despite modest implementation costs. Findings support economic feasibility of low‐technology, mechanically focused oral care strategies	Level C

The evidence synthesis was conducted narratively, organized around predefined analytic domains corresponding to the review objectives such as pathophysiologic mechanisms along the oral–lung axis; current oral care practices; modality‐specific interventions; assessment and documentation; implementation and workforce factors; safety, equity, and feasibility, prioritizing the most recent and methodologically robust reviews where overlap existed and using earlier studies to assess consistency overtime. Key characteristics and findings of the included studies are summarized in Table 1.

## 4. Discussion

Oral care is widely promoted as a core component of pneumonia prevention measures in both VAP‐ and NVHAP‐hospitalized patients. Emerging evidence has improved understanding of the oral–lung axis and combined with evolving clinical interventions has expanded the scope of bedside oral care from basic hygiene to a structured preventive practice referring to a standard set of oral hygiene and infection–prevention measures delivered systematically as part of routine patient care. However, there are still significant uncertainties about optimal oral care practices, their independent effects on clinical outcomes, and the most effective implementation models. This limits the clear attribution of benefit to specific oral hygiene components and hinders translation into standardized practice. It is important to identify these knowledge gaps to inform future research on more practical and patient‐centered approaches.

### 4.1. Pathophysiological Rationale Linking Oral Health to VAP and NVHAP

The development of hospital‐acquired pneumonia in critically ill adults is closely linked to rapid and profound alterations in the oral environment that occur early during ICU admission. Critical illness disrupts normal salivary flow, mucosal integrity, and microbial homeostasis, allowing dental plaque and oropharyngeal biofilms to shift toward pathogenic communities capable of colonizing the lower respiratory tract. Mechanical ventilation further introduces artificial surfaces and secretion reservoirs that promote bacterial proliferation and migration. Together, these processes establish a biologically plausible oral–lung axis through which oral hygiene becomes a modifiable target for interrupting both VAP and NVHAP.

#### 4.1.1. Dental Plaque and Oropharyngeal Biofilms

In healthy adults, dental plaque and salivary defenses maintain a relatively stable oral ecosystem. During critical illness and mechanical ventilation, this balance deteriorates rapidly. Within 48 h of ICU admission, plaque accumulation increases, gingival inflammation worsens, and potentially pathogenic respiratory organisms colonize dental surfaces and the oropharynx [25, 34]. Endotracheal tubes provided additional substrates for biofilm formation. Bacteria embedded within these biofilms may be dislodged into the lower airway or migrate along the endotracheal cuff, particularly when cuff pressures are suboptimal or secretions are allowed to pool above the cuff [35–37]. Experimental and clinical studies have demonstrated concordance between microbial species isolated from dental plaque, oropharyngeal secretions, and lower respiratory tract samples in patients who develop VAP, supporting the oral cavity as an important reservoir for pneumonia pathogens [38, 39].

#### 4.1.2. Aspiration Pathways and Impaired Defense

Sedation, neuromuscular blockade, reduced consciousness, and supine positioning impair cough and swallowing reflexes, increasing the likelihood that colonized oral secretions are aspirated into the trachea [40, 41]. Secretions that accumulate above the endotracheal cuff are particularly relevant; when not removed, they may leak into the lower airway during positional changes or ventilator adjustments, substantially increasing the infection risk [42].

Importantly, the aspiration risk is not confined to mechanically ventilated patients. Dysphagic, bedbound, and postoperative patients in step‐down units and acute wards are also vulnerable to NVHAP when oral care is infrequent and mobility is limited [43, 44]. Reduced saliva production caused by medications, oxygen therapy, and open‐mouth posture further promotes plaque accumulation and fungal overgrowth, compounding the infection risk.

#### 4.1.3. Key Opportunities for Care

From a mechanistic perspective, oral care can intervene at multiple points along this pathway.

• Physical plaque removal: Toothbrushing or equivalent mechanical methods reduce plaque burden and biofilm thickness, limiting bacterial load and pathogenic shifts, although evidence linking brushing alone to pneumonia reduction remains inconsistent [19, 45].
• Disinfection: Antiseptic agents such as chlorhexidine (CHX) and alternative mouthwashes aim to suppress bacterial growth and delay recolonization; however, recent evidence has questioned their net clinical benefit and safety when used routinely [46].
• Secretion management and suction: Low‐volume rinsing combined with immediate suction facilitates the removal of dislodged debris and contaminated saliva before aspiration occurs [47].
• Mucosal hydration: Regular moisturizing helps preserve mucosal integrity, reduce fissuring and discomfort, and may limit secondary fungal infection, although direct effects on pneumonia outcomes are poorly defined [48].
• Device and denture care: Cleaning of oral devices and dentures and minimizing mucosal pressure points reduce sites for bacterial growth and epithelial injury that may serve as portals for infection [49, 50].

### 4.2. Current Oral Hygiene Practices in Critical Care Settings

Across ICUs internationally, oral hygiene practices display significant variability driven by differences in training, resource availability, and institutional culture. Although oral care is widely recognized as essential, its delivery is frequently inconsistent, with documented gaps in assessment, brushing frequency, tool selection, and adherence to structured protocols. These patterns highlight a persistent disconnect between evidence‐based recommendations and bedside practice, underscoring the need to understand current behaviors before evaluating improvement strategies.

Survey and observational data consistently show a gap between what research shows works best and how bedside oral care is performed [21, 51]. Work from the United Kingdom and other regions in Europe has shown that although most ICU nurses report performing some form of oral assessment, only a minority use organized assessment tools [21, 52]. Similar gaps are recorded in Asian ICUs, where foam swabs and saline are still common, and the frequency of documented oral care is often lower than planned targets [13, 53, 54].

A national point‐prevalence study in adult ICUs reported that only about two‐thirds of ventilated patients were covered by a written oral care procedure and just under three‐quarters had documented oral care in the preceding 24 h [21]. Fewer than half had received toothbrushing, and CHX mouthrinse was used in a small minority [21]. Studies from other countries point to similar inconsistencies and a continued dependence on foam swabs despite limited evidence that they offer advantages over toothbrushes for plaque removal [31, 34, 50, 55].

At the same time, NVHAP prevention programs have shown that oral care is often absent or not recorded on general wards, and many patients who develop NVHAP have no recorded oral care in their electronic records [6, 31, 32]. These findings match national recommendations to make practice consistent, make oral care a key safety focus, and include it in hospital pneumonia‐prevention routines [12, 56].

Overall, current practice is marked by incomplete follow‐up of procedures, variations in tools and frequency, and inconsistent recording, even though most clinicians recognize the importance of oral hygiene.

### 4.3. Effects of Oral Hygiene Interventions

Multiple therapeutic approaches have been used to limit the oral microbial burden and aspiration risk in critically ill adults, ranging from mechanical plaque removal to chemical disinfection and secretion management. Each intervention targets a distinct aspect of the pathogenic cascade that contributes to VAP and NVHAP. Evaluating the evidence supporting these methods is essential to determining their relative contributions, the conditions under which they are effective, and the degree to which they modify clinically meaningful outcomes.

#### 4.3.1. Mechanical Plaque Removal (Toothbrushing)

Various research findings support toothbrushing, or an equivalent mechanical method, as the essential practice [10, 19, 34]. Randomized trials comparing different structured oral hygiene plans show that adding toothbrushing to usual oral care reduces dental plaque scores and bacterial growth, with suggestions of reduced VAP in some studies, although most are modest in size [7, 16, 42]. The 2020 Cochrane review of oral hygiene care in critically ill adults concluded that protocols including both antiseptics and toothbrushing may be more effective for VAP and ICU LOS than antiseptics alone, though the certainty of evidence was low [9].

Regardless of additions, the consistent message across reviews and practice guidelines is that mechanical plaque removal is the essential foundation of ICU oral care, ideally using a soft and small‐headed toothbrush, with attention to all tooth surfaces and the gingival margin [7, 57]. Tongue cleaning, especially when heavily coated, is practical, though specific data on pneumonia outcomes are limited [58].

#### 4.3.2. Antiseptic Mouthwashes and Alternatives

The role of antiseptic mouthwashes has been changed. Earlier trials and meta‐analyses suggested that CHX mouthrinse or gel reduced VAP incidence without affecting death rates, ventilator days, or overall ICU LOS [20, 27]. Concerns about potential harm emerged when some studies reported a possible mortality signal in certain populations, particularly cardiothoracic surgery patients, though whether it caused this remained uncertain [58]. Recent studies have reinforced this uncertainty. A network meta‐analysis did not demonstrate a consistent advantage of CHX over alternative oral care strategies for preventing VAP, and the overall certainty of evidence was rated low to moderate [29]. A recent network meta‐analysis that synthesized randomized trials up to 2024 found that combinations involving brushing plus 0.12% CHX ranked highest for VAP prevention, followed by CHX alone and brushing alone, while acknowledging low certainty for most comparisons [11]. Another network meta‐analysis ranked toothbrushing combined with CHX among the higher‐performing interventions; however, this finding was accompanied by wide confidence intervals and low confidence ratings [30].The growing agreement is that standard use for everyone of CHX for all ventilated patients is not well supported, particularly given unanswered safety questions and the shift toward broader HAP prevention as a priority [28, 30, 59]. Instead, targeted use in specific patient groups such as cardiothoracic or high‐risk surgical patients appears reasonable as several trials and meta‐analyses demonstrate VAP reduction in these specific groups [60, 61].

For units that stop using CHX, the evidence base for other disinfectants remains limited. Recent pediatric and adult work suggests that antiseptic‐based treatment plans do not consistently improve major outcomes such as death rates, time on breathing machines, or ICU stay, even when VAP is diagnosed; this emphasizes the need that antiseptics should be viewed as optional additions, not as replacements for brushing and suction [9, 62].

#### 4.3.3. Suctioning and Secretion Management

Oral care that physically cleans plaque but permits loosened germs and particles and bacteria‐filled saliva to collect above the cuff misses an important opportunity. Currently available evidence consistently stresses using low‐volume water or solution rinses followed by immediate suction, ideally with suction toothbrushes or separate Yankauer suction, to clear secretions from the oropharynx and subglottic region [7, 57].

Where subglottic suction endotracheal tubes are available, integrating routine suction cycles into oral care can help limit the buildup of secretions and may contribute to VAP reduction as part of broader ventilator routines. High‐quality trials separating the suction method and timing from other components are limited, and most data comes from multielement VAP‐prevention packages, so linking the benefit to any single component remains uncertain [63, 64].

#### 4.3.4. Moisturization and Mucosal Care

Research studies confirm that oral mucosal integrity deteriorates during ICU stays, with increasing dryness, redness, sores, and bacterial growth over the first days of admission, especially in patients on multiple medications and with low Glasgow Coma Scale scores [65, 66]. While direct links between how often moisturizing is done and pneumonia outcomes remain limited, moisturization every 2–4 h using nonirritant gels or water‐based products is supported for comfort, preventing cracks, and possibly reducing secondary infections.

Given the lack of high‐quality comparative trials, product selection should prioritize safety, ease of application, and working well with other oral care elements rather than theoretical antimicrobial advantages alone.

#### 4.3.5. Denture, Device, and Interface Hygiene

For edentulous patients or those with partial dentures, cleaning and appropriate storage of dentures are critical. Dentures act as biofilm carriers and can be reservoirs for respiratory pathogens if they are not brushed and soaked regularly [67, 68]. Oral tissue damage from medical equipment like nasogastric tubes, oxygen interfaces, and bite blocks can also damage protective tissues and trap bacteria. Regular inspection, repositioning, use of protective dressings, and early involvement of dental professionals can reduce these risks [38, 69].

Although high‐quality VAP outcome trials focused on denture care alone are lacking, including denture and device hygiene into oral care procedures is affordable and consistent with established mechanisms of infection and match and microbial colonization [70, 71].

#### 4.3.6. Special Critical Care Populations

• Cardiothoracic ICUs: Earlier studies suggested that antiseptic oral care might have a stronger mortality finding in cardiac surgery populations, but updated research reviews have questioned the strength of this effect once trial quality and other treatments are considered [72–74].
• Tracheostomy care: Long‐term tracheostomy patients have distinct bacterial growth patterns and mucus buildup. Frequent mouth and throat suction, stoma hygiene, and ongoing oral care remain relevant, but high‐quality data for this patient group are limited [75].
• Limited‐resource settings: Reviews from low‐ and middle‐income countries show greater reliance on makeshift tools, limited toothbrush availability, and fewer antiseptic products, but the same basic processes apply. Simple, low‐cost routines focused on twice‐daily brushing, clean water rinses, suctioning where available, and basic moisturization are likely to offer substantial benefits [76, 77].

Data gaps are common across these contexts. Many patient groups lack trials that focus on oral care intensity or specific methods, so recommendations often rest on biological logic and general critical care basics.

### 4.4. Oral Health Assessment and Documentation

Accurate recognition of oral changes in critically ill patients is fundamental to guiding care intensity and detecting the deterioration of oral health. However, oral status is often poorly assessed and inconsistently recorded despite rapid shifts in plaque accumulation, mucosal breakdown, and secretion pooling during critical illness. Structured oral health checks in mechanically ventilated adults remain limited and are not used consistently [25]. Existing tools typically rate plaque, gingiva or mucosa, tongue, and saliva, but only a minority of studies report formal agreement between different assessors, and most have not been tested specifically for use by ICU nurses or linked directly to pneumonia outcomes [25].

Studies from adult ICUs show wide variation in whether oral assessments are performed at all, how they are recorded, and how often oral care is delivered [14]. Prevention programs for NVHAP that have reported reductions in pneumonia rates have structured oral care elements, clear recording spaces, and continuous tracking with feedback to frontline teams [22, 23] (Table 2).

**Table 2:** Oral assessment and documentation workflow for mechanically ventilated adults.

Phase	Assessment/action	Components
Admission oral assessment	Initial oral evaluation	First shift after ICU admission or intubation; record dentition/dentures, plaque, mucosa, tongue, saliva, bleeding risk; set initial brushing and moisturization plan
Routine reassessment	Ongoing monitoring	At least twice daily with nursing shifts; reassess plaque, mucosa, tongue coating, dryness/secretions; adjust care plan; refer if ulcers, bleeding, or infection suspected
Ordinal scoring	Risk stratification	Score plaque, mucosa, tongue, saliva on a 0–3 or 1–4 scale; use scores to flag patients requiring senior review
Documentation	Clinical recording	Enter data in structured EHR fields; record care actions (brushing, suction, moisturizer, antiseptic); note reasons if care is missed or modified
Feedback and training	Quality improvement	Aggregate adherence, scores, and HAP events; use findings in feedback sessions and targeted training

*Note:* A proposed workflow [21–23, 25, 56].

Training in oral assessment and oral care procedures, including education, supervised practice, and feedback, has been associated with higher nursing skills, improved oral care delivery, and, in some studies, better oral health and lower incidence of VAP [78–81]. However, data from large surveys indicate that oral care is still not consistently included as a required skill in ICU nursing training programs [82–84].

### 4.5. Implementation Challenges and Workforce Readiness

Sustained improvement in oral hygiene practices centers on the ability of ICU teams to prioritize, operationalize, and consistently deliver evidence‐based care within complex clinical environments. Competing workload demands, supply inconsistencies, knowledge gaps, and variable role clarity influence the feasibility of routine oral care. Understanding these organizational and behavioral constraints is critical to designing interventions that are both practical and resilient under real‐world conditions. Knowledge, attitude, and practice studies consistently show that ICU nurses recognize the importance of oral care but face barriers including time pressure, competing priorities, inconsistent supplies, and uncertainty about best techniques [81, 83–85]. Oral care is often delegated to less‐experienced staff or treated as a task that can be deferred when the workload is high.

Core implementation strategies include:

• A ready‐made kit was kept at the bedside containing a soft toothbrush, suction‐compatible device, moisturizer, and any approved antiseptic. Ready‐to‐use kits reduce effort and inconsistency and have been part of several successful pneumonia prevention programs [55, 84].
• Clear roles and timing, assigning oral care clearly within each shift’s task list and ventilator bundle, and clarifying which team members own which components [81].
• Skills‐based training included hands‐on practice, use of assessment tools, and clear teaching about pneumonia causes. Training programs focusing on oral care can improve knowledge and reported practice, though direct links to patient outcomes are less often measured [85, 86].
• Monitoring and reporting using simple measures such as percentage of ventilated patient days with recorded brushing twice daily or NVHAP events per 1000 patient days broken down by recorded oral care [81, 83].
• A team approach across various specialties, bringing dentists and dental hygienists, infection control staff, and ICU clinicians together to design procedures, select products, and review harmful events [84, 85].

Where these elements are aligned, ICU and hospital‐wide programs have reported lasting reductions in NVHAP and associated poor outcomes, although many reports are observational studies that may be affected by other factors [55, 85].

### 4.6. Vital Role of Dentistry and Oral Health Professionals

From a dental and oral health perspective, hospitalized patients, particularly those in critical care, constitute a high‐risk population for rapid oral biofilm maturation, periodontal inflammation, mucosal injury, and microbial dysbiosis. Reduced salivary flow, impaired self‐care, denture use, xerostomia, and prolonged hospitalization accelerate oral deterioration within days of the admission. These changes are clinically consequential rather than localized: seminal ICU studies have demonstrated that dental plaque and oropharyngeal biofilms frequently harbor respiratory pathogens concordant with those isolated from the lower airways of patients who develop hospital‐acquired pneumonia, identifying the oral cavity as an important reservoir for infection [87]. Epidemiological evidence further associates periodontal disease and poor oral health with increased risk of nosocomial pneumonia, reinforcing oral health as a determinant of respiratory outcomes in both VAP and NVHAP [88].

Within this context, integration of dental expertise represents a realistic expansion of hospital oral care protocols. While nurse‐led oral care remains central to routine prevention, dentistry‐informed input can enhance structured assessment, risk stratification, and targeted intervention in patients with complex oral diseases that exceeds the scope of standard bedside care. Hospital‐based evidence supports this complementary role. A quasi‐experimental ICU study by Sabino et al. [89] demonstrated that a dentist‐led, multidisciplinary oral health protocol was associated with substantial reductions in ventilator‐associated events and VAP‐related mortality among mechanically ventilated patients, underscoring the value of structured dental leadership in high‐risk cohorts. Similarly, a 6‐year retrospective ICU analysis by Pains et al. [90] found that greater exposure to dental experts in the ICU was independently associated with lower mortality without increased adverse events. Collectively, these findings support embedding dental and oral health professionals within hospital pneumonia‐prevention pathways through defined consultation triggers, standardized assessment frameworks, and coordinated delivery of mechanically focused oral care, complementing rather than displacing established nurse‐led bundles.

### 4.7. Safety Considerations

Providing oral care in critically ill adults requires careful attention to patient‐specific risks arising from coagulation abnormalities, agitation, airway vulnerability, and device dependence. Inadequate technique or inappropriate tool selection can result in aspiration, mucosal trauma, or device‐related injury, each of which can compound clinical instability. Incorporating safety principles into oral hygiene practices is therefore essential to minimizing preventable harm while achieving therapeutic goals.

Reported harmful events include equipment problems, oral tissue damage, breathing in fluids or objects, and disinfectant side effects. Foam tip breaking off and breathing in the foam have been highlighted in UK and international medical device alerts and agreed guidelines, with cases of choking and death prompting clear warnings about their use (Medicines and Healthcare products Regulatory Agency [MHRA], 2012; British Association of Critical Care Nurses [BACCN], 2020) [91, 92]. They have noted both the risk of breathing in foam pieces and the limited cleaning ability of foam swabs compared with toothbrushes and have recommended moving toward toothbrush‐based or otherwise proven tools [92].

Oral tissue injury and bleeding are concerns in patients with low platelet counts, clotting disorders, or severe mouth sores, where standard brushing forces or stiff tools can cause sores or bleeding. In such patients, procedures generally call for softer tools, reduced force and frequency, and temporary pausing of brushing when there is active bleeding. Breathing in fluids during oral care is another risk when large fluid volumes are used without coordinated suctioning or when patients are poorly positioned or unable to protect their airway; low‐volume rinses, coordinated suction, and head‐of‐bed elevation are routine safety measures in critical care guidelines and oral‐care agreed statements [92].

Key harmful events, typical scenarios, and practical prevention methods are summarized in Table 3. Consistent recording of oral care‐related harmful events remains uncommon; integrating such events into routine safety reports and oral care quality tracking is important to improve procedures and reassure staff that safety is being closely watched [91, 92].

**Table 3:** Safety considerations and adverse events associated with oral care in critically ill adults.

Adverse event	Typical scenario	Prevention/Response
Foam swab detachment/aspiration	Foam head loosens; patient bites or coughs	Prefer toothbrushes or approved devices; inspect swabs before use; avoid in highly agitated patients
Mucosal trauma/bleeding	Thrombocytopenia, coagulopathy, severe mucositis	Define platelet/coagulation cut‐offs; use ultra‐soft brush or gauze; reduce force/frequency; temporarily defer if active bleeding
Aspiration during oral care	Large‐volume rinses, poor positioning, weak suction	Use low‐volume rinses; coordinate with suction; keep head elevated; stop if cough or distress occurs
Antiseptic‐related effects	Chlorhexidine or other antiseptics in high‐risk patients	Use only when clearly indicated; follow local concentration/indication policy; monitor for allergy or intolerance; report suspected serious harm

*Note:* Reported adverse events from literature are summarized with typical scenarios and corresponding prevention/response strategies (e.g., MHRA [91]; Klompas et al. [20]; De Cassai et al. [29]).

### 4.8. Equity, Cost, and Feasibility

The practicality of delivering high‐quality oral care varies across healthcare systems, influenced by staffing ratios, resource constraints, and institutional priorities. Approaches that are effective in well‐resourced ICUs may not be scalable in settings with limited supplies or high patient‐to‐staff ratios. Ensuring equitable pneumonia prevention requires identifying low‐cost and adaptable strategies that preserve effectiveness, minimize the workflow burden, and support consistent implementation across diverse clinical contexts. Pneumonia‐prevention programs that rely on specialized commercial products, frequent monitoring, or large education budgets may be difficult to sustain in smaller or limited‐resource ICUs [24, 33, 93].

At the same time, the essential elements of effective oral care are low‐tech: soft toothbrushes, clean water or basic solutions, simple moisturizers, and suction. Reviews from various resource levels support the view that consistent twice‐daily brushing with suction, combined with regular moisturizing and head‐of‐bed elevation, can be delivered without expensive products and is likely responsible for much of the observed pneumonia risk reduction [80, 94].

Equity also includes record‐keeping demands. Complex electronic reminders risk discouraging complete recording in critically ill patient units. Minimal, useful data points aligned with quality measures are more realistic. Finally, pneumonia‐prevention efforts must include non‐ICU wards, where staffing ratios are tighter and oral care is easier to overlook, despite a significant pneumonia burden [24, 95].

### 4.9. A Practical Oral Hygiene Bundle for Mechanically Ventilated Patients

Integrating evidence from physiological mechanisms, clinical trials, and real‐world implementation supports the development of a streamlined, reproducible oral hygiene routine for mechanically ventilated adults. A structured bundle clarifies essential steps, aligns care across teams, and reduces practice variability. By standardizing assessment, brushing, secretion control, moisturization, and selective antiseptic use, this approach transforms fragmented bedside actions into an organized prevention strategy.

**Figure 2:**
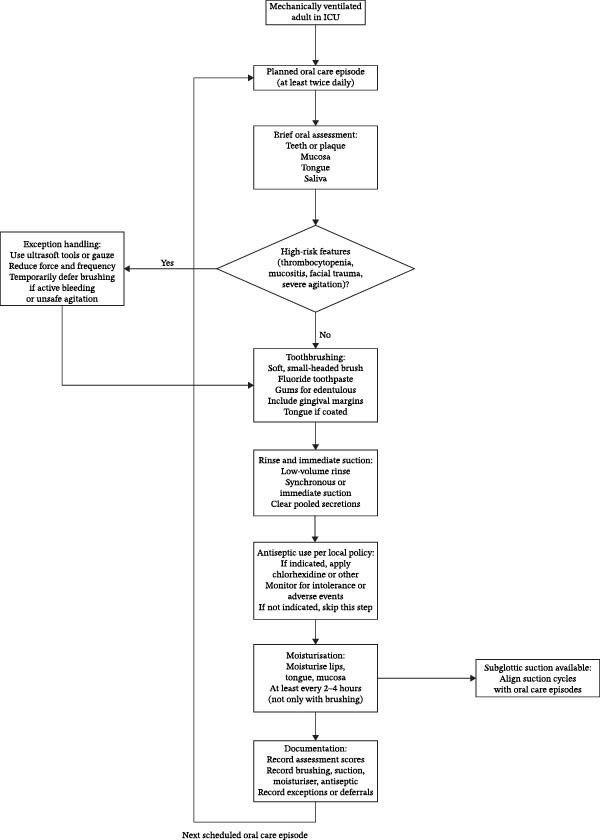
Practical oral care bundle and bedside algorithm for mechanically ventilated adults.

Based on the preceding evidence, this review proposes a practical oral care routine that can be delivered as a repeating cycle each shift. The routine includes a systematic oral assessment, twice‐daily toothbrushing with low‐volume rinse and immediate suction, regular moisturization, and targeted antiseptic application according to local policy, with modifications for patients at high bleeding risk, those with mucosal injury, facial trauma, or severe agitation, and integration with subglottic suction where available. Figure 2 summarizes this process in a simple flowchart.

The step‐by‐step flowchart summarizes a twice‐daily oral care routine focused on systematic oral check‐ups, toothbrushing, low‐volume rinse with immediate suction, frequent moisturization, targeted antiseptic use according to local policy, coordination with subglottic suction where available, and clear adjustments for patients with bleeding risk, sores, facial injuries, or severe agitation, with all elements documented in electronic health records.

This routine can be adjusted across settings by changing the product choice and documentation depth rather than changing the main steps of brushing, suction, moisturization, and assessment.

### 4.10. Evidence Gaps and Research Priorities

1. Brushing vs. antiseptics and suction: Older and newer research studies agree that oral care packages reduce VAP incidence, but separating the independent effects of brushing, antiseptics, and suction is difficult because most trials combine these elements. Analyses of combined studies suggest brushing (with or without CHX) ranks highly, while recent work questions the benefit on its own of CHX [96, 97].
2. Mortality and LOS effects: Even when VAP incidence falls, clear, consistent effects on mortality, duration of mechanical ventilation, and total LOS are harder to demonstrate. This may reflect diagnostic variation in VAP definitions, other influencing factors, and the influence of other care processes [9, 26, 46].
3. Generalizability of cardiothoracic findings: Earlier patterns from combined studies of mortality benefit from antiseptic oral cleaning were heavily driven by cardiothoracic patient groups. Whether these findings extend to general ICU populations is doubtful, particularly given the updated analyses of combined studies and concerns about CHX‐associated harm [20, 61, 72].
4. Clinical outcomes: Many studies report improvements in plaque scores, gingival measures, or indirect bacterial growth measurements without a strong connection to VAP or NVHAP. The degree to which changes in oral scores translate into fewer pneumonias, shorter stays, or better functional outcomes remains not fully measured [18, 19, 98].
5. Diagnostic variation: Differences in diagnostic thresholds, radiographic criteria, and microbiologic sampling complicate combining results and may hide or overstate the apparent impact of oral care [6, 64].
6. Limited evaluation of dentistry‐led models: Despite strong biological plausibility and emerging hospital‐based evidence, the contribution of dental professionals to structured oral assessment, targeted intervention, and team‐based oral care remains insufficiently evaluated in VAP and NVHAP prevention trials. Dentistry‐led oral health protocols have been associated with reductions in ventilator‐associated events and mortality in ICU settings but have not been systematically tested using standardized pneumonia definitions or implementation‐focused study designs across diverse hospitals [89, 90]. These gaps support the need for more focused, well‐designed trials and real‐world testing rather than implying that oral care is unimportant.

Emerging evidence underscores the need for a shift in research priorities. Further small explanatory trials that compare marginal product substitutions are unlikely to resolve the key uncertainties identified in this review. What is now required are adequately powered, pragmatic, cluster‐randomized, or stepped wedge studies that isolate the incremental value of brushing intensity versus antiseptic choice and suction strategy; use standardized, transparent definitions for VAP and NVHAP; report patient‐centered outcomes, including functional recovery, discharge destination, and quality of life, alongside traditional ICU metrics. Within this agenda, dentistry‐led oral assessment frameworks and interprofessional oral care models including defined roles for dental and oral health professionals represent a critical but underexplored research direction, particularly for patients with complex oral disease or prolonged hospitalization.

Oral health assessment tools need formal validation in mechanically ventilated populations, with calibration, interrater reliability reporting, and explicit linkage between ordinal scores, colonization patterns, and pneumonia events. Implementation studies should treat oral care as a complex behavior embedded within workload, staffing, and supply constraints using theory‐informed strategies, equity‐sensitive designs, and cost‐effectiveness analyses across ICU and non‐ICU wards. Systematic surveillance of oral care‐related adverse events, including foam swab complications and mucosal injury in high bleeding‐risk patients, should be built into quality dashboards rather than left to sporadic device alerts.

As with any integrative review, the inherent methodological constraints should be considered when interpreting these findings. This review was restricted to English‐language publications with full‐text availability, which may have excluded relevant international data. Considerable heterogeneity existed in intervention components, diagnostic definitions of VAP and NVHAP, and reported outcomes, limiting comparability across studies. Additionally, reliance on narrative synthesis may introduce interpretive bias despite structured evidence appraisal.

## 5. Conclusion

Oral hygiene occupies a tractable point along the oral–lung axis where simple bedside actions can modify pneumonia risk, yet practice remains heterogeneous and under‐specified. Evidence supports the idea that structured oral care packages reduce VAP incidence, particularly when brushing and suction are reliably delivered, even though the effects on mortality, duration of ventilation, and LOS remain more uncertain. NVHAP initiatives extend this logic to general wards, where the same mechanisms apply but oral care is even less consistently documented or audited.

Taken together, the existing data support the use of twice‐daily mechanical plaque disruption with a soft toothbrush, combined with low‐volume rinses and immediate suction, as the key component of ICU oral care. Moisturizing and mucosal care, denture hygiene, and structured oral assessments are low‐cost additions that address xerostomia, biofilm reservoirs, and documentation gaps without relying on proprietary products. Antiseptic agents, particularly CHX, are better viewed as optional adjuncts that may be appropriate for defined surgical or high‐risk cohorts rather than universal prophylaxis, given the mixed mortality signals and absence of consistent benefit on hard outcomes. For policy and practice, a reasonable approach based on current evidence is the use of a parsimonious, reproducible bundle: twice daily brushing with suction, frequent moisturizer, head of bed elevation, basic device care, and minimal high‐yield electronic fields that support audit and feedback.

If these clinical, methodological, and implementation gaps are addressed, oral care can move from a variably executed comfort task to a clearly specified, auditable component of hospital‐acquired pneumonia prevention. The central message is not that oral care alone can eliminate VAP or NVHAP but that consistently delivered, low‐technology oral care, aligned with broader ventilator and mobility practices, is a reliable and scalable contribution to safer critical and acute care.

## Author Contributions

Chris Sara Mathew, Manjush Karthika, and Khalid Ansari contributed to conceptualization and design, acquisition, analysis, interpretation of data, drafting, and critically revising.

## Funding

This study is self‐funded, and no external funding is received.

## Ethics Statement

This integrative review was based exclusively on previously published studies and publicly available data. No primary data were collected, and no human participants or identifiable personal information were involved. Therefore, ethical approval and informed consent were not required for this study.

## Conflicts of Interest

The authors declare no conflicts of interest.

## Data Availability

The study‐level data used to support the findings of this integrative review are included within the article, including the evidence summary tables and narrative synthesis. No new datasets were generated.
